# The oral microbiome and salivary proteins influence caries in children aged 6 to 8 years

**DOI:** 10.1186/s12903-020-01262-9

**Published:** 2020-10-28

**Authors:** Wang Chen, Qian Jiang, Guowei Yan, Deqin Yang

**Affiliations:** 1 College of Stomatology, Chongqing Medical University, Chongqing, China; 2 Chongqing key Laboratory of Oral Diseases and Biomedical Sciences, Chongqing, China

**Keywords:** Dental caries, Oral microbiota, Salivary proteins, 16S rDNA, iTRAQ

## Abstract

**Background:**

Oral microbiome and salivary proteins play a critical role in the occurrence and development of caries. In this study, we used metagenomic and metaproteomic analyses to explore the microbiological and proteinic biomarkers and investigate the etiology of caries in 6–8 years old children. Our study aims to offer a better comprehension of these factors and the relationship with caries, and these findings might facilitate caries risk assessment and provide a basis for future prevention strategies.

**Methods:**

Children 6 to 8 years old living in rural isolated areas including 40 caries-active subjects and 40 caries-free subjects were recruited. Supragingival plaque and unstimulated saliva were collected for 16S rDNA pyrosequencing and isobaric tags for relative and absolute quantitation (iTRAQ) technique coupled with quantitative nano-flow liquid chromatography-tandem mass spectrometry (LC-MS/MS), respectively.

**Results:**

We found 6 phyla and 13 genera predominant in all the samples, and differences in relative abundances can be observed. The Alpha diversity analysis demonstrated that the richness and diversity of the bacterial communities were similar between children with caries-free and caries-active groups; LEfSe detected differences in the bacterial community including Dialister, Selenomonas, Actinomyces, and Mogibacterium in the caries-active group (*P* < 0.05) and Capnocytophaga, Fusobacterium, Desulfuromonadales, Haemophilus, and Porphyromonas in the caries-free group(*P* < 0.05). The core microbiome was defined as 18 predominant genera in children with caries. The results of the salivary proteome identified 9135 unique peptides and 1662 proteins group from 20 salivary samples. Two hundred fifty-eight proteins were differentially expressed between the caries-free and caries-active groups.

**Conclusions:**

The diversity of the microbial community has little effect on caries but some bacteria with different relative abundance between the caries-active and caries-free group could be considered as potential biomarkers for children with caries. In addition, as a critical host factor of caries, the salivary proteins are different in caries-free and caries-active groups.

## Background

It is estimated that 2.4 billion people suffer from dental caries, and 621 million of them are children [1]. Severe caries can affect their quality of life [2]. Strategies to prevent caries are based on a comprehensive understanding of its etiology and effective control of the risk factors. It is recognized that causes of caries include microorganisms in the mouth and host factors. The oral cavity is one of the most diverse and complex microbial environments [3]. Previous studies demonstrated that oral plaque film has high relevance in dental caries. The acid produced from bacteria break the balance of tooth mineralization and demineralization and the host have no rapid response to pH changes, which lead to organic degradation [4]. Saliva is the main microenvironment of oral microorganisms, and to some extent, saliva microorganism determines the structure of plaque. Salivary protein has a crucial role in monitoring health status or monitoring disease [5]. It was reported that the proteins in saliva could modulate the balance of oral health and homeostasis, maintain a stable ecosystem, and inhibit the growth of cariogenic bacteria [6].

In the past few decades, several investigators have proposed several hypotheses regarding the etiology of caries [7–9], the relationship between bacteria and dental caries, the complexity of the oral bacterial structure, and the difference of bacterial components. Previous studies also mentioned that some salivary proteomic molecules could regulate the oral cavity microbial flora and correlate to caries [6, 10, 11]. Unfortunately, due to differences in samples, technologies, and analytical methods, the results remain controversial and the biomarker information unclear.

Thanks to recent advancements in molecular biology techniques, metagenomic and metaproteomic can be used to obtain a complete analysis of the oral bacteria and proteomic. Next-generation sequencing technologies have been successfully applied in oral microbial analysis [12–14]. The isobaric tags for relative and absolute quantitation (iTRAQ) is a new technique which uses isotopes to label polypeptides for comparing proteomes quantitatively [15, 16]. To the best of our knowledge, previous studies of caries-related microbiome and proteome were detached. Our present study uses metagenomic and metaproteomic analyses to explore the microbiological and proteinic biomarkers and investigate caries etiology in children.

In this study, we enrolled 6–8 years old children (isolated population) who come from Tujia and Miao minority autonomous county, Pengshui, Chongqing, China. These children have a simple and homogeneous diet; therefore, the impact of different diets and daily living habits is avoided. In the current study, the oral microecological diversity was studied using 16S rDNA pyrosequencing, and the salivary proteins were analyzed using the iTRAQ technique coupled with quantitative nano-flow liquid chromatography-tandem mass spectrometry (LC-MS/MS). Our study aimed to 1) detect the microbiological compositions and to investigate the core microbiome; 2) identify the salivary proteomic and characterize the functional classification in children with or without caries, and 3) attempt to identify microbiological or proteinic biomarkers helpful to prevent dental caries.

## Methods

### Subjects selection and sample collection

All the study participants were recruited from primary schools located in Tujia and Miao minority autonomous county, Pengshui, Chongqing, China, in February 2014. This area is a remote mountainous area whose population has a simple diet and low mobility. Before enrollment, parents or guardians of the subjects (6–8 years children) were provided with informed consent explaining the study objectives. According to the criteria from the World Health Organization, 4th-edition publication of “Oral Health Surveys, Basic Methods,” children whose dmfs (missing due to caries, or filled tooth surfaces in primary teeth) index was over eight were divided into a caries-active group, and caries-free group (dmfs = 0). Finally, 40 caries-active subjects (20 males and 20 females), and 40 caries-free subjects (20 males and 20 females) were selected. All the children have 1) no long-term (> 3 months) history of living in different places; 2) no antibiotic therapy; 3) no use of fluoride at least 3 months before the examination; and 4) no other oral diseases or systemic diseases [17]. This study was approved by the Ethics Committee of Affiliated Hospital of Stomatology of Chongqing Medical University (the date of approval was 1/1/2012 and the approval number is CQHS-IRB-2016-05).

Supragingival plaque and unstimulated saliva from the 80 subjects selected were collected in the morning before eating, drinking, and tooth brushing. All the subjects were required to rinse their mouth with sterile water for 30 s before sample collection. Caries-active plaque was collected from each caries site, and caries-free plaque was collected from healthy molar surfaces. The samples were placed in 1.5 ml sterile Eppendorf tubes. Unstimulated saliva was collected into the sterile plastic cups (excluding sputum), and then transferred to 5 mL EP tube after 5 min. All the samples that were mixed with blood and other residues were discarded. Samples were immediately transported to the laboratory on ice and stored at − 80 °C in the laboratory until further processing.

### Oral microbiome analysis

#### DNA extraction and purification

The genome of all the samples was extracted using Promega Genomic DNA Purification Kit, following the manufacturer’s instructions. To detect if the sample was free from contamination, 5 μl DNA samples were taken for agarose gel electrophoresis (110 V, 20 min). We selected distinct bands showing no obvious trailing phenomenon, which showed that the genome was relatively complete without significant RNA and protein contamination. Then we evaluate the quality by measuring the absorbance at A260/280 using a UV spectrophotometer (DU-800, Beckman Coulter). The samples with the A260: A280 ratios at 1.8:2.0, and the DNA concentrations in 20–100 ng/μl were screened, and the results indicated that the genomic DNA extracted met the requirements for subsequent sequencing [18]. Finally, after screening 40 high-quality samples [13, 19] were selected to perform sequencing analysis: SN (caries-free saliva group, *n* = 10); PN (caries-free plaque group, *n* = 10); SH (caries-active saliva group, *n* = 10); and PH (caries-active plaque group, *n* = 10). The DNA samples were stored at − 20 °C before use.

#### PCR amplification and pyrosequencing

The general primers for PCR amplification of the bacterial 16S rDNA V1-V3 region were the reverse primer 533R (5′-TTACCGCGGCTGCTGGCAC-3′), and forward primer 8F(5′-AGAGTTTGATCCTGGCTCAG-3′). After adding the tag sequence, the 454 Life Science A or B sequencing adaptor was connected with general primers by linker sequence [20]. PCR amplification was performed using Trans Start Fastpfu DNA Polymerase (TransGen AP221–02), three replicates per sample. The PCR products of the same sample were taken for 2% agarose gel electrophoresis, and the AxyPrep DNA Gel Extraction Kit was used to recover the PCR products. Afterward, the 16S rRNA gene was sequenced on the Roche 454 GS FLX+ Sequencing Method Manual_XLR70 kit.

#### Bioinformatics analysis

Ambiguous base, homologous base, and sequences shorter than the original 200 bp sequence were removed or discarded to obtain high-quality sequences [21]. The high-quality sequences (≥ 80% confidence) were compared using the SILVA database [22] (version106) and Mothur software (version 1.31.2) [23] at a 97% similarity level. Based on the results of operational taxonomic units (OTUs) clustering analysis; community richness and diversity indices of ACE, Chao, Shannon, Simpson, and the Good’s coverage were calculated. We constructed a circle phylogenetic tree using the ITOL platform to explore the relationships of the general microbial population. The principal coordinates analysis (PCoA) was based on Bray-Curtis distances at an OUT level with 97% identity. PCoA was used to compare the similarities in the bacterial community structures among the four groups. The linear discriminant analysis (LDA) of effect size (LEfSe) was performed to define the biological class features and establish statistical significance [24]. A Venn diagram was made using Mothur software to reveal the core microbiome. The significant differences in microbial community composition were analyzed using one-way ANOVA with SPSS Software (version 25.0), and statistical significance was set at *P* < 0.05.

### Salivary proteomics analysis

#### Sample preparation

Salivary samples from the SN and SH groups used in the metagenomic analysis were selected for further proteomics analyses. A total of 20 saliva proteome samples from each group were pooled (SN = 10, SH = 10), and the mixture was centrifuged in 5 KDa ultrafiltration tube for concentration until the volume was about 200 μL. Protein quantification was performed using the Bradford assay with bovine serum albumin (BSA) as standard and analyzed with SDS-PAGE. Twenty picograms of protein sample were mixed with SDS-PAGE sample loading buffer (10% SDS, 0.5% BTB, 50% glycerinum, 500 mMDTT, 250 mM Tris HCl pH 6.8) in a ratio of 1:5 v/v, incubated in a boiling water bath for 5 min and then centrifuged at 14000 g for 20 min. The supernatant was taken for 12.5% SDS-PAGE electrophoresis (14 mA, 90 min).

#### Proteins filter-aided sample preparation (FASP)

The method of filter-aided sample preparation (FASP) was used for protein extraction, digestion, and peptide separation. Samples from the SN and SH groups were mixed with SDT Lysis Buffer (4%SDS,100 mM Tris-HCl,1 mM DTT pH 7.6), incubated in a boiling water bath and then centrifuged in 30KDa ultrafiltration tube to a final volume of 25 μL. To remove large excess of detergent and interfering substances, UA buffer (8 M urea,150 mM Tris HCl pH 8.0) was mixed with protein extract in 30 KDa ultrafiltration tube and centrifuged at 14000 g for 15 min. The filtered liquor was discarded, and the on-filter remaining material was added 100 μL IAA (50nmM IAA in UA) and centrifuged at 14000 g for 10 min. This process of extensive washes and buffer exchange was repeated several times. The peptides were quantified using OD280.

#### iTRAQ labeling and SCX fractionation

Ninety picograms of treated samples from the SN and SH groups were labeled with the iTRAQ Reagent-4plex Multiplex Kit (AB SCIEX) according to the manufacturer’s instructions. Peptides from each group were labeled with the following tags: 114 and 116 tags for the SN, 115 and 117 tags for SH, respectively. Each labeled peptide segments were mixed an underwent a strong cation-exchange chromatography (SCX) fractionation. The SCX gradient information is provided in the additional Table 1. According to the SCX chromatogram, ten fractions were combined, which then were lyophilized and desalinated using C18 Cartridge (Sigma-Aldrich, St Louis, MO, USA).

#### Mass spectrometry analysis

Peptides were loaded to the Thermo scientific EASY column (2 cm × 100 μm 5 μm-C18) and then separated using the same Thermo scientific EASY column (75 μm × 100 mm 3 μm-C18) mounted in an EASY-nLC 1000 system with the flow rate of 250 nl/min. Buffer A consisted of 0.1% formic acid, while buffer B consisted of 0.1% formic acid, 84% ACN. The chromatographic column was balanced with 95% buffer A. The flow rate of the gradient started at 0% buffer B, going to 35% buffer B in 100 min, continuing to 100% buffer B in 8 min, and maintaining 100% buffer B in 120 min.

The eluates were injected into a Q-Exactive mass spectrometer (Thermo Fisher Scientific, Waltham, MA, USA), run in positive ion mode with a full MS scan from 300 to 1800 m/z. The MS/MS spectra acquisition parameters were as follows: full scan resolutions set to 70,000 at m/z 200; the AGC target was 3 × 106 with a maximum fill time of 10 ms; dynamic exclusion set to 40 s. We used higher collision energy dissociation (HCD) to collect the mass-charge ratio of peptide fragments. Ten MS2 scans were collected after each full scan. The normalized collision energy (NCE) was 30 eV.

#### Data analysis

The raw data were processed using Proteome Discover 1.3 (Thermo Fisher Scientific, Waltham, MA, USA; version 1.3) and searched against the International Protein Index human database (ipi. Human.v3.87.fasta) containing 91,464 sequences using the Mascot search engine (Matrix Science, Boston, MA, USA; version 2.2). The additional Table 2 shows the parameters used for the database search. Proteins were filtered, and the false discovery rate (FDR) of peptide and protein level was less than 1%. The ionic peak strength values of peptides were quantitatively analyzed with the Proteome Discoverer 1.3(Thermo Scientific, San Jose, California, USA). The student’s t-test was used to evaluate the differences between the two groups, which were considered statistically significant if P<0.05. Proteins with quantification *P*-value < 0.05 and fold changes > 1.2 were identified as differentially expressed proteins. Functional classification of differentially expressed proteins was evaluated performing gene ontology (GO) analysis, which includes three-term of biological processes, molecular function, and cellular components.

## Results

### Plaque and salivary microbiome

#### Sequences information and bacterial diversity

All the samples were divided into four groups. After 454 pyrosequencing, a total of 415,203 16S rRNA sequences were obtained from 40 samples, 20 from plaque and 20 from saliva, and 328,486 high-quality sequences (79%) passed the quality-control test. The mean sequence length was 476 bp, with an average sequencing depth of 6347 reads per sample. All the qualified sequences (≥ 97% similarity level), were compared to the SILVA database (version10.6) using Mothur software (version 1.31.2) and the reads were clustered into 14,076 operational taxonomic units (OTUs) including 6042 OTUs from caries plaque, 6757 OTUs from caries-free plaque, 5406 OTUs from caries saliva, and 5561 OTUs from caries-free saliva (Table 1).

**Table 1:** Statistics of the Microbial Number in Plaque and Saliva from Several Taxonomic Levels

	PH	PN	SH	SN
Phylum	13	15	12	14
Class	20	23	20	21
Order	33	35	35	36
Family	50	52	62	54
Genus	82	90	105	97
OTU(0.03)	6042	6757	5406	5661

The indices of Shannon, Simpson, Chao, and ACE were calculated to obtain the bacterial richness and diversity; the Good’s coverage reflects the sequencing depth. The oral microbial diversity parameters are shown in Table 2; there was no significant difference in the richness and diversity of the bacterial communities between caries and caries-free group(*P* > 0.05). The Good’s coverage for each group was over 95%, indicating adequate sequencing depth. Chao curve and Shannon curve (Additional Fig. 1) show the change of bacterial richness and diversity with the increase of sequencing numbers. When the number of reads approached 15,000, the curve became flat, which indicated that the number of sequences was appropriate to reflect the microbial information. The rarefaction curve was plotted (Additional Fig. 2) to reflect the adequacy of the number of sequences used to obtain the desired number of OUT. The result showed that with the increase of sequencing number, the trend of OTU quantity was up, but the latter was not flat enough. This result indicated that microorganisms of plaque and saliva were rich, and rarer species could be discovered if the sample size increases.

**Table 2:** Community Richness Estimator and Diversity Estimator of Every Group

	PH	PN	SH	SN
ACE (0.03)	18587 (18044,19155)	20059 (19502,20641)	14641 (14189,15117)	16707 (16198,17241)
Chao (0.03)	12126 (11549,12764)	12813 (12273,13406)	10159 (9686,10685)	10919 (10410,11482)
Shannon (0.03)	6.74 (6.73,6.75)	6.8 (6.78,6.82)	6.53 (6.51,6.54)	6.54 (6.53,6.56)
Simpson (0.03)	0.0038 (0.0037,0.0039)	0.0034 (0.0033,0.0035)	0.0049 (0.0049,0.005)	0.0045 (0.0044,0.0046)
Coverage (0.03)	0.951656	0.964732	0.959671	0.958081

#### Bacterial community structure and composition in different niches

**Fig. 1:**
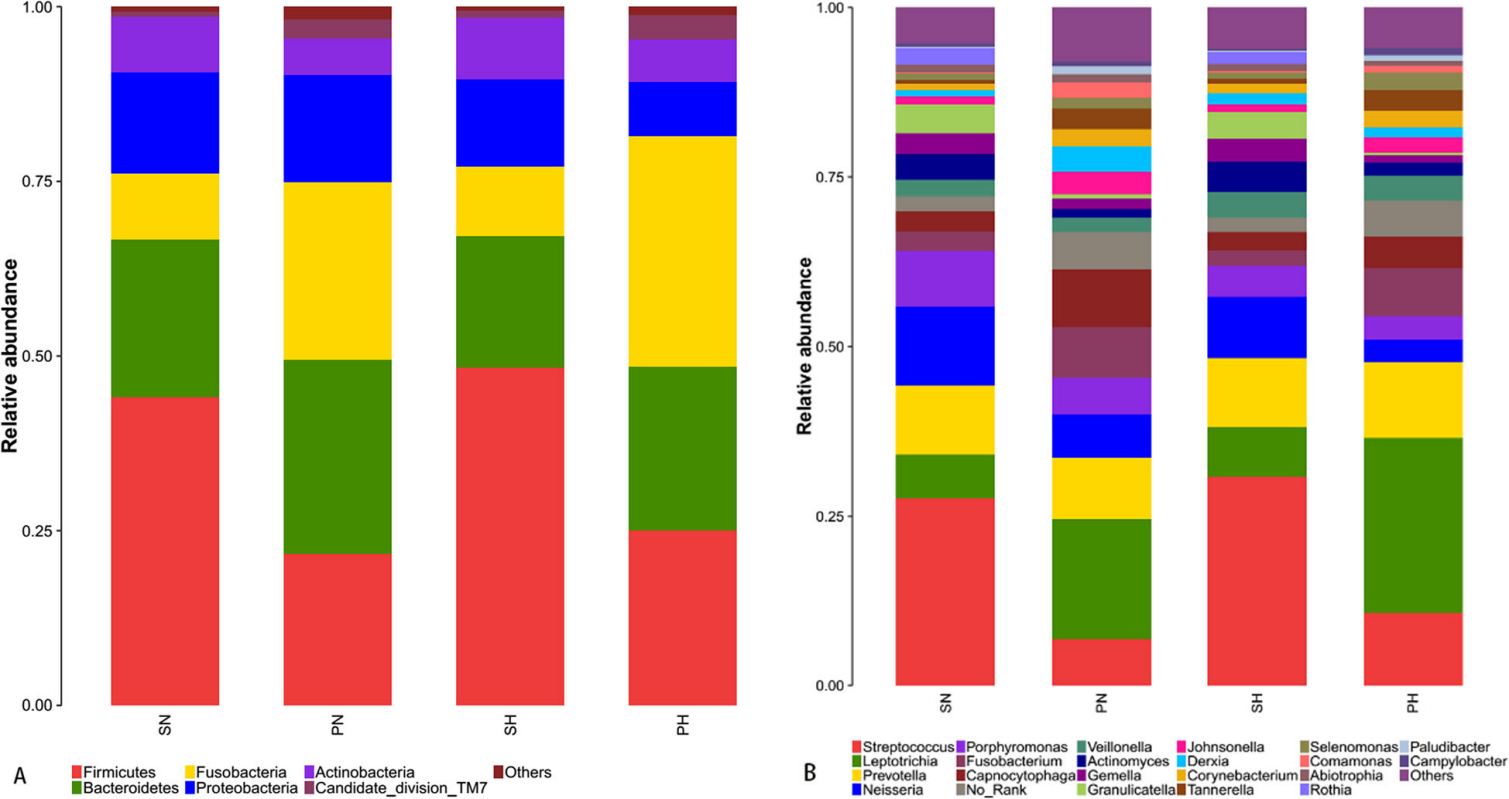
The Distributions of Predominant Taxa at the Phylum and Genus Level in Each Group. The distributions of predominant taxa (relative abundance > 2% on average) on **a** phylum and **b** genus level

**Fig. 2:**
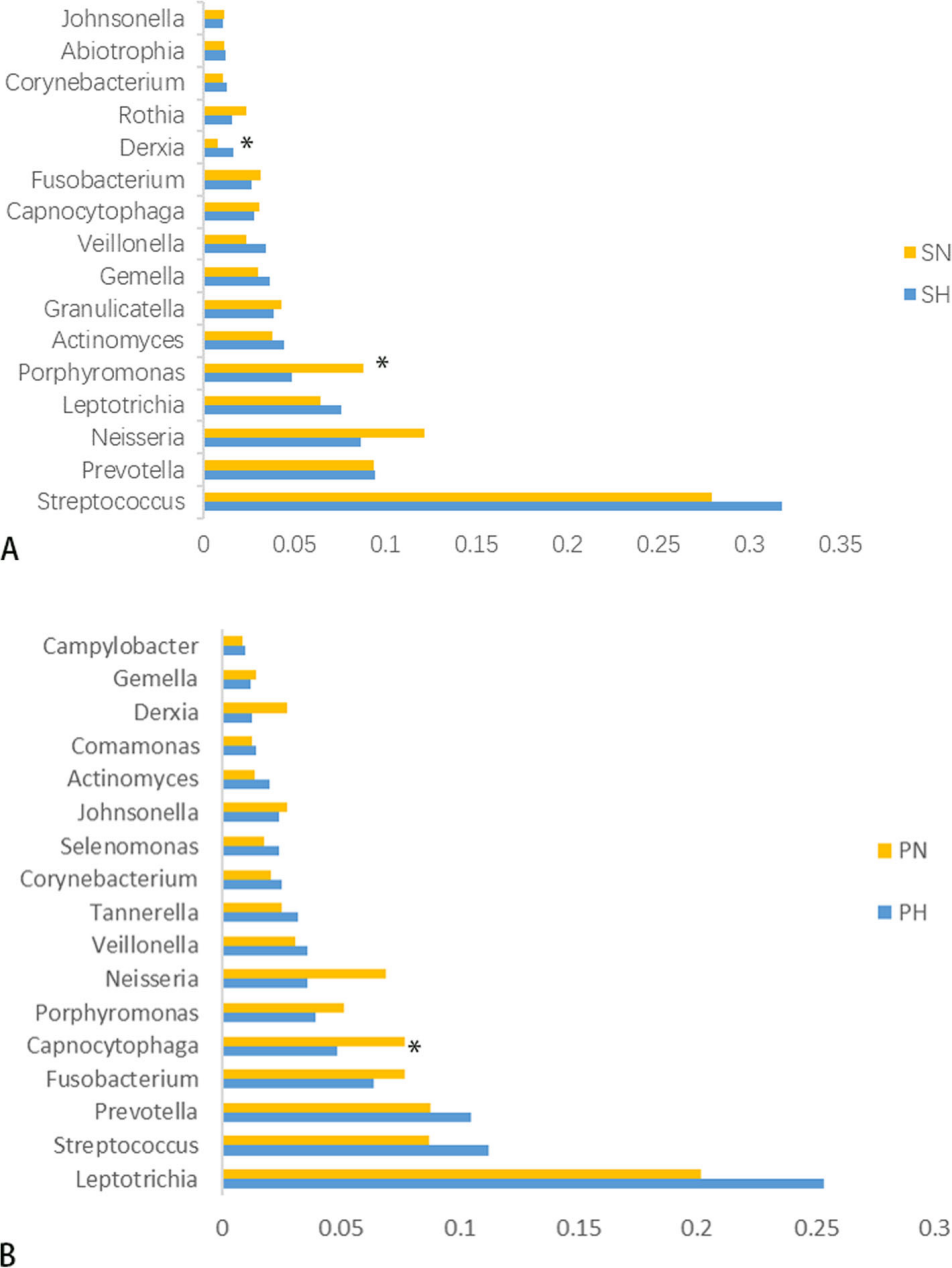
The Relative Abundance Comparison of the Predominant Bacteria at the Genus Level. Wilcoxon rank-sum test analyzes the difference. * represents a significant difference (*P* < 0.05)

A total of 18 phyla, 28 classes, 48 orders, 78 families,135 genera, and 410 species were detected in the 40 samples analyzed. Overall, the six most abundant phyla were Firmicutes (33.66%), Bacteroidetes (23.61%), Fusobacteria (19.83%), Proteobacteria (12.89%), Actinobacteria (6.85%), and Candidate division TM7(2%). Together, they represent 98.84% of the total sequences. The reads were dominated by 13 genera including Streptococcus (17.86%), Leptotrichia (14.60%), Prevotella (9.99%), Neisseria (7.51%), Porphyromonas (5.47%), Fusobacterium (5.12%), Capnocytophaga (5.10%), Veillonella (2.86%), Actinomyces (2.79%), Gemella (2.20%), Granulicatella (2.17%), Johnsonella (2.09%), and Derxia (2. 06%). They occupied 79.84% of the whole. Figure 1a and b show the taxonomic distributions of the predominant bacteria at the phyla and genera levels. The relative abundance between caries-active and caries-free subjects was compared using the Wilcoxon rank-sum test. The significant difference was detected among four groups (additional Table 3 and additional Table 4). A higher abundance (relative abundance > 1%)of Porphyromonas was detected in the SN group(*P* = 0.044) and Derxia in the SH group(*P* = 0.045). A higher abundance (relative abundance > 1%) of Capnocytophaga was observed in the PN group than the PH group(*P* = 0.019)(Fig. 2).

Since Actinomyces and Streptococcus are acknowledged to play important roles in dental caries, they were particularly analyzed at species level. As shown in Additional Fig. 3, Actinomyces odontolyticus was the most abundant Actinomyces species, followed by Actinomyces gerencseriae. For Actinomyces odontolyticus, there was no significant difference between the caries-free and caries-active groups. While Actinomyces gerencseriae was detected to be more abundant in the caries-active group (*P* < 0.05). The difference of Streptococcus at the species level is shown in the Additional Fig. 4. Streptococcus sanguinis was the most abundant Streptococcus species and it was higher in caries-free plaque than caries-active plaque. Streptococcus cristatus was the second abundant Streptococcus species, accounting for more than 0.5% of all the bacterial species.

**Fig. 3:**
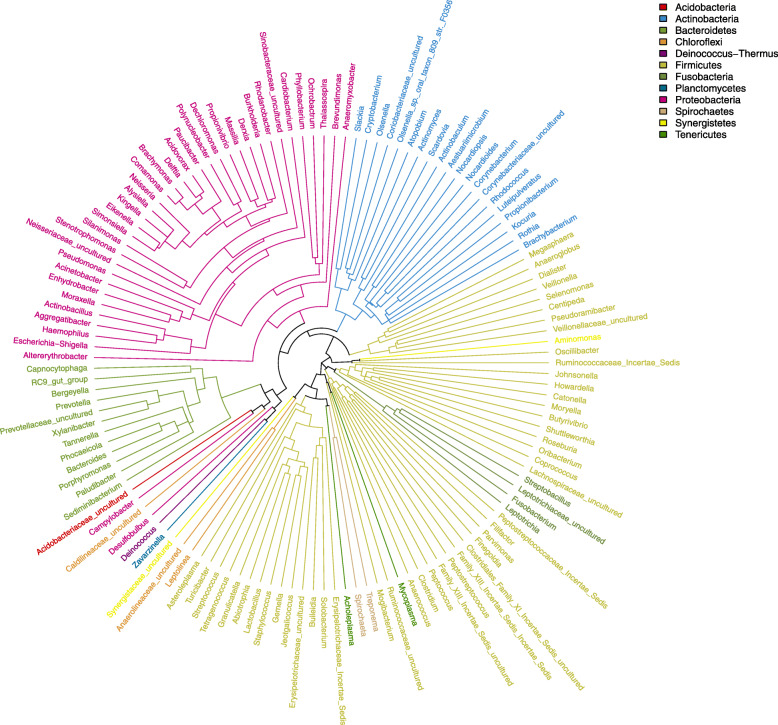
Circular Phylogenetic Tree at the Level of the Total 133 Genera. The tree was generated with Fasttree. The color-coded branches from the inner to the outer circles corresponds to the taxa from the phylum to genus level based 12 abundant phylum

**Fig. 4:**
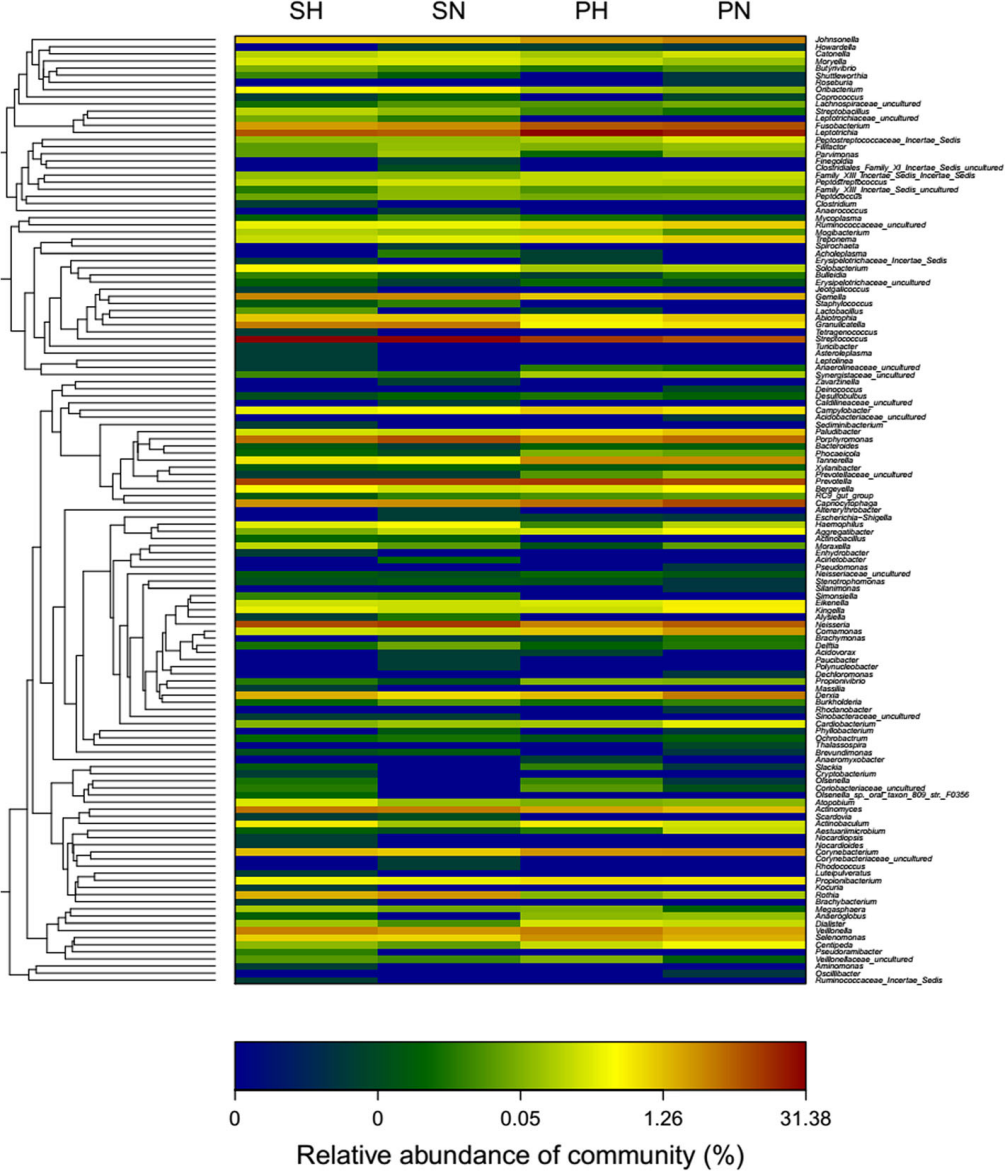
Relative Abundance of the 133 bacterial Genera in Each Group. Each column represents one genus of the groups. The relative abundance (%) is indicated according to the color scale at the bottom of the plot

To explore the relationship of the bacterial-community, a circular phylogenetic tree from 133 genera was constructed (Fig. 3). The relative abundance in caries-free and caries group, as well as the community composition at the genus level, could be observed in the Heatmap (Fig. 4), in which we could find that the predominant microbial communities were largely similar, but the variety of individual microorganism is apparent among the four groups.

**Fig. 5:**
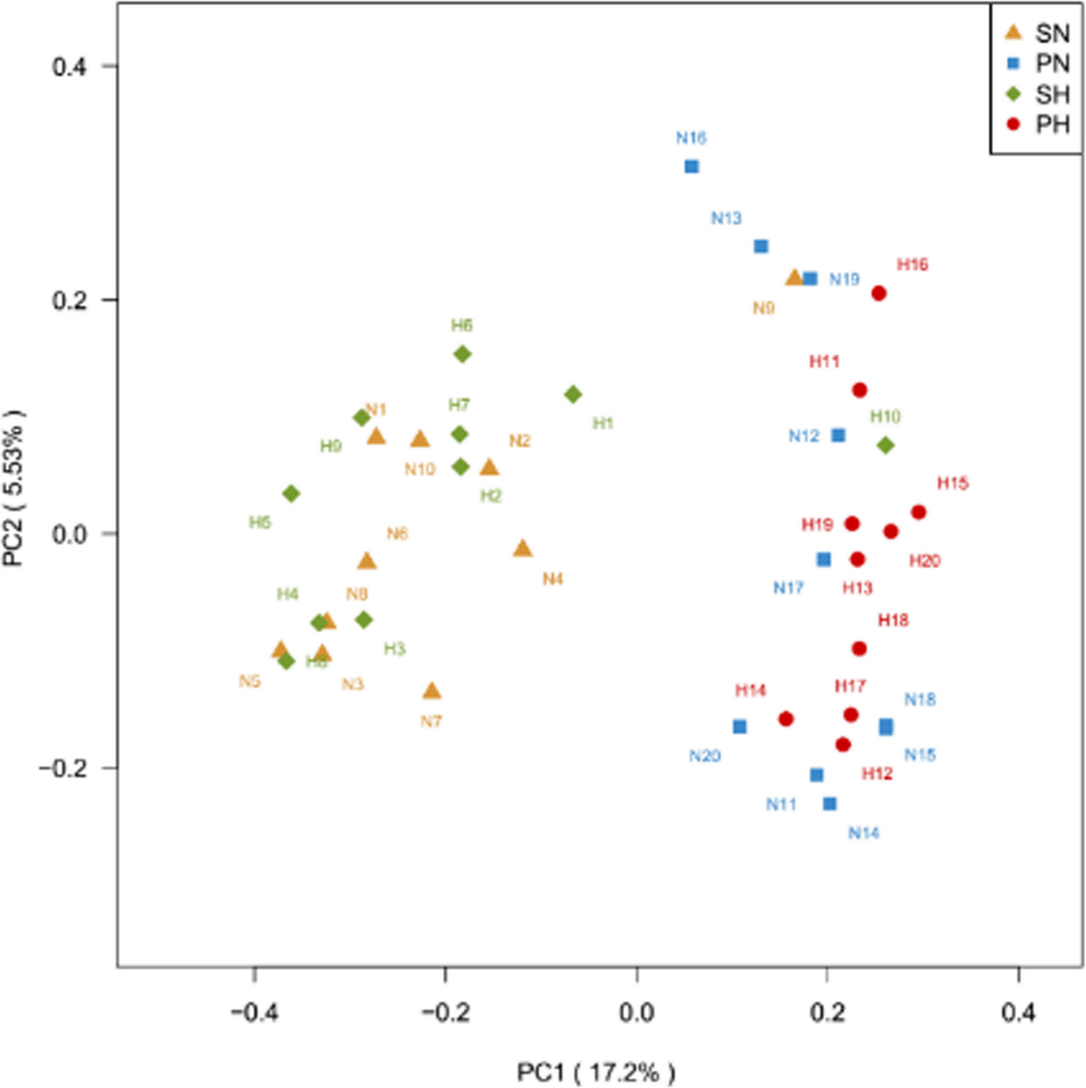
PCoA based on the Bray-Curtis Distances at OUT level with 97%.A dot represents each sample. Orange triangles represent the SN samples. Green rhombus represents the PN samples. Blue squares represent the PN samples. Red balls represent the PH samples. PC1 explained 17.2% of the variation observed, while PC2 explained 5.53% of the variation

The analysis of similarities (ANOSIM) was performed to compare the relatedness of microbiome composition among the four groups [25]. The principal coordinates analysis (PCoA), based on the Bray-Curtis distances, demonstrated segregations between samples from dental plaque and saliva. As shown in Fig. 5, the microbiota from the SN group overlapped with SH, so as PN and PH groups. A clear difference can be observed within the saliva and the plaque samples. The PCoA result indicated that the oral microbial community compositions are similar between caries-active patients and healthy controls; nevertheless, there were some dissimilarities in two different niches. These differences were also observed using the nonmetric multidimensional scaling (NMDS) analysis (Additional Fig. 5).

**Fig. 6:**
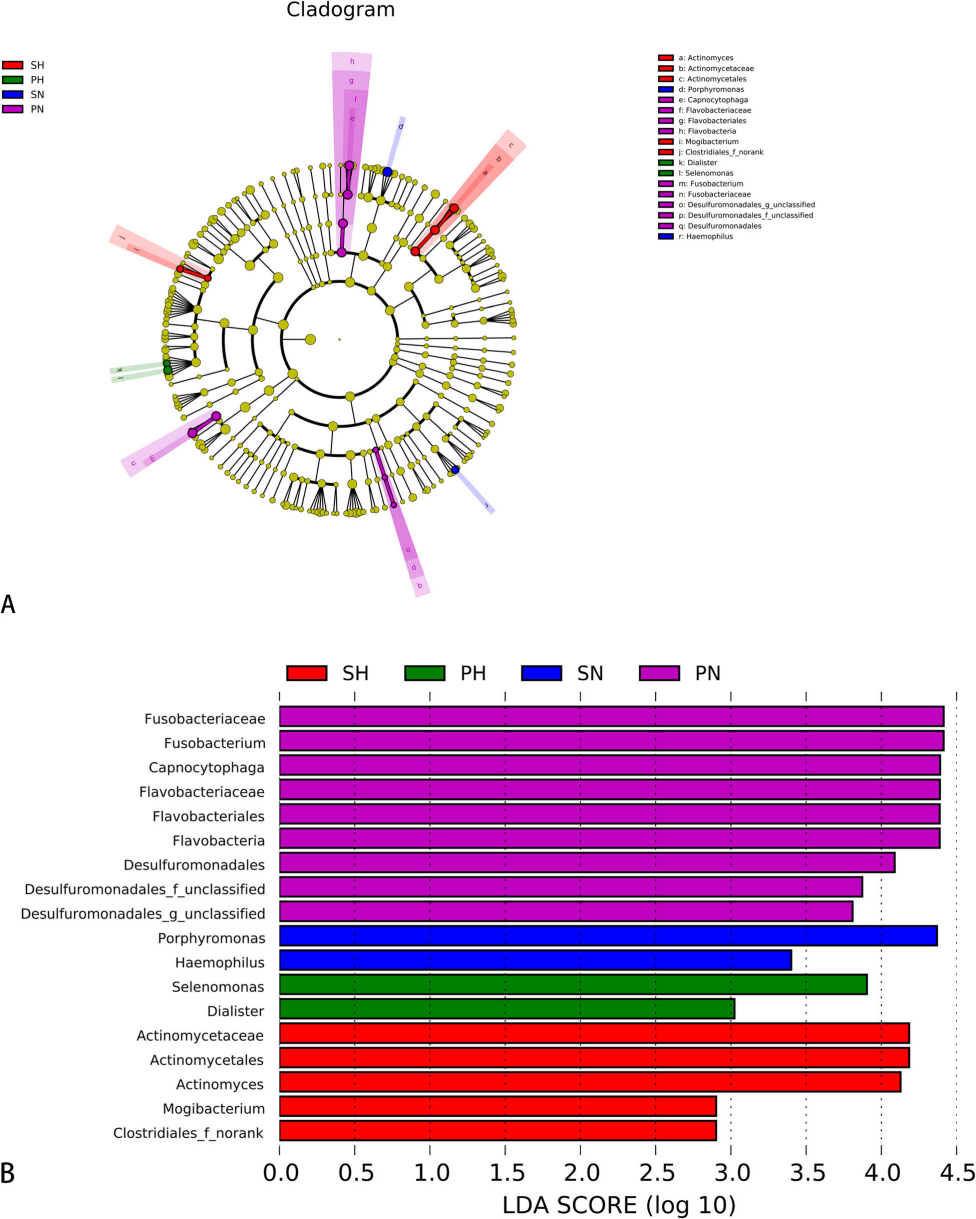
LEfse Analysis Based on LDA Shows the Microbial Variations. **a** Cladogram for the taxonomic representation of the significant differences among the four groups. The colored nodes from the inner to the outer circles represent taxa from the phylum to genus level. The colorized point represents the different taxa with more a significant role. **b** Histogram of the LDA scores: the higher the LDA score, the greater the species impact on the different effects. The threshold on the logarithmic LDA score for discriminative features was set at 2.0

The LEfSe analysis was performed to expose differences in the bacterial community composition, which later could be regarded as the biomarkers of different groups, and used to identify potential caries-related and health-related bacteria [26], and their effect sizes were represented in a taxonomic tree. Figure 6a shows cladograms representing the microbial community with significant differences at different levels. Significant differences detected in the PH group were Dialister and Selenomonas at the genus level (*LDA* > 2, *P* < 0.05). Capnocytophaga Fusobacterium and Desulfuromonadales exhibited relatively higher abundance in the PN group (*LDA* > 2, *P* < 0.05). As for the saliva group, Actinomyces and Mogibacterium were significantly enriched in the SH group (*LDA* > 2, *P* < 0.05), while the relative abundance of Haemophilus and Porphyromonas was higher in the SN group (*LDA* > 2, *P* < 0.05). Figure 6b shows the LDA score representing the impact of differential features among groups.

#### The core microbiome

**Fig. 7:**
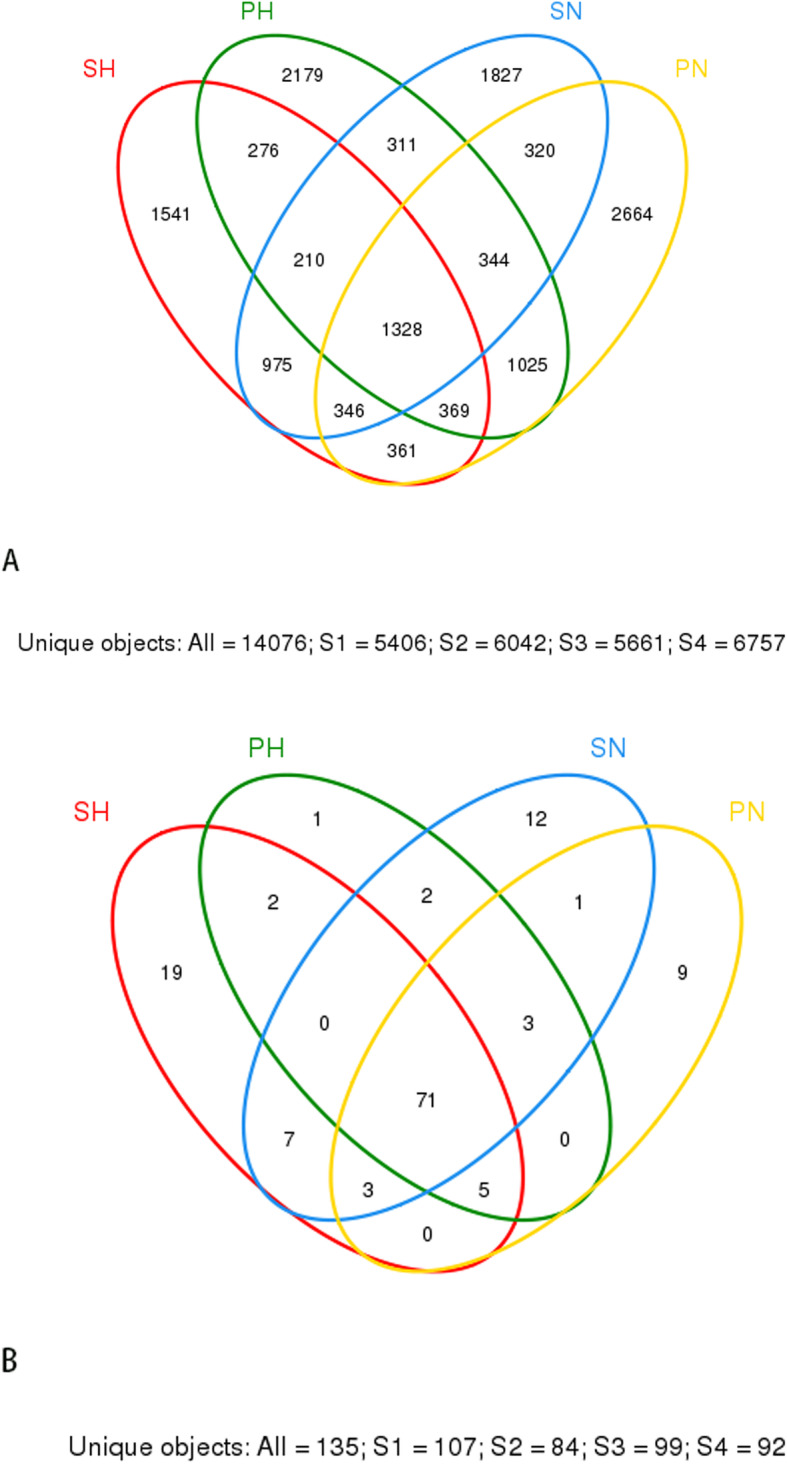
A Venn Diagram Showing Shared (A) OTUs and (B) genera with 97% Identity

A Venn diagram was used to display the core microbiome, the overlapping areas in the circles stand for the members shared among the four groups in each taxonomical level. The oral microbiome analysis revealed an overlap of shared OUTs and genera. As the Venn diagram shows (Fig. 7a,b); 14,076 OTUs were identified including 5406, 6042, 5661, and 6757 OTUs in the SH, PH, SN and PN groups, respectively. A total of 1328 OTUs and 71 genera were common among the four groups, occupying 9.4% of all the OTUs (14,076 OTUs), and 52.6% of all the genera (135 genera) detected. We detected 18 predominant genera uniform in the samples from saliva and plaque subjects including Abiotrophia, Actinomyces, Bergeyella, Campylobacter, Capnocytophaga, Corynebacterium, Derxia, Fusobacterium, Gemella, Granulicatella, Johnsonella, Neisseria, Porphyromonas, Prevotella, Propionibacterium, Streptococcus, Veillonella, and Ruminococcaceae uncultured. This shared microbiome supports the existence of an “oral core microbiome” and this shared genera may be part of the oral core microbiome in the dental plaque and saliva of caries from patient and healthy controls.

### Differentially expressed proteins related to dental caries and its functional classification

Proteomic analysis of saliva samples was performed using the iTRAQ-coupled LC-MS/MS method to detect protein biomarkers of caries risk in children. Two saliva samples from the SN (caries-free saliva group, *n* = 10) and SH (caries-active saliva group, *n* = 10) were used for this study. The protein bands of the saliva samples from subjects with and without caries were not entirely consistent with SDS-PAGE electrophoresis (Additional Fig. 6), indicating the existence of differentially expressed salivary protein between healthy and cariogenic children. The salivary protein samples from the SN and SH groups were also used (90 μg for each group) for the iTRAQ analysis. After querying the database, a total of 9135 unique peptides and 1662 proteins group (unique peptides ≥1) were identified, including 1626 proteins with quantitative information (Additional file 13). Pearson correlation between each experimental group and its replicate showed good reproducibility (Additional Fig. 7a,b). We found 258 proteins to be differentially expressed according to the criteria of *P*-value < 0.05 and ratio- fold change > 1.2. Some differential expressed proteins between caries and healthy saliva were listed in the additional Table 5, *such as lactoferrin, mucin, the family of matrix metalloproteinase and cystatin, immunoglobulin peptides, protein S100, and proline-rich protein* and so on*.* All of these were considered to be associated with dental caries.

**Fig. 8:**
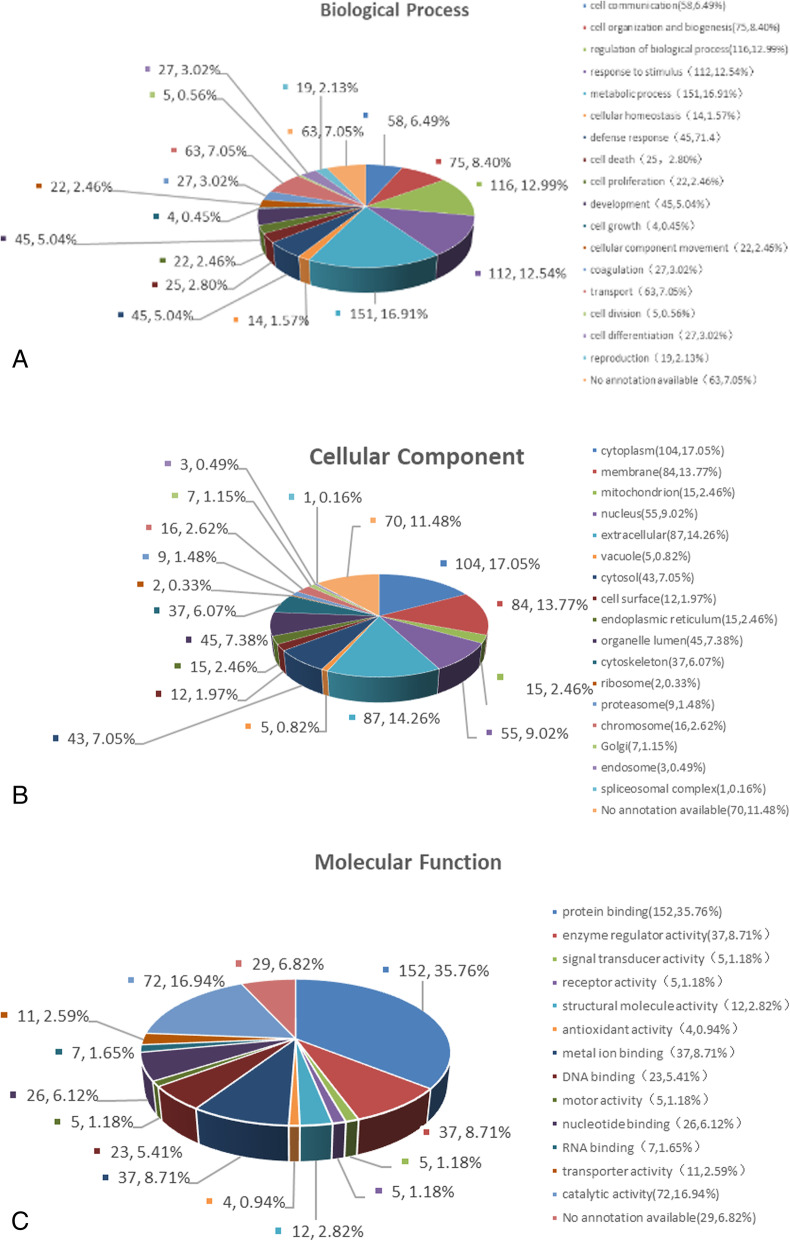
Gene Ontology Analysis of the Differentially Expressed Proteins. **a** biological processes **b** cellular components **c** molecular function

Gene ontology analysis was performed to explore the biological function of the differentially expressed proteins base on their biological processes, molecular function, and cellular components. The proteins involved in the metabolic process (16.91%), regulation of biological process (12.99%), and response to the stimulus (12.54%) were enriched in the SH group compared with healthy controls (Fig. 8a). The majority of differentially expressed proteins were found in the cytoplasm (17.05%), extracellular (14.26%), and membrane (13.77%) (Fig. 8b). GO analysis showed that proteins involved in the protein binding (35.76%) and catalytic activity (16.94%) were enriched in the SH group (Fig. 8c).

## Discussion

The etiological concepts of oral infectious diseases, including caries and periodontal disease, has gradually changed from a single pathogen theory to a microecological imbalance theory [4, 27, 28]. Therefore, a systems biology approach is required to explain the complex interactions between the microbiome and the host. As far as we know, an approximate of 1000 bacterial species have been found in the oral cavity [29] due to the advent of molecular analysis methods. Recently, 16S rRNA sequence analysis was introduced in the study of uncultured oral microbial communities; this is an advantageous molecular analysis technology for investigating the oral bacteria diversity and microbial community composition in oral diseases. Meanwhile, salivary proteins play an essential role in the occurrence and development of caries. Proteomics has advanced significantly over the past decades, and it has been applied for the study of caries and other oral diseases [28, 29]. In this study, we preliminarily explored microbiome and host factors in childhood caries using the high-throughput technique of 16S rDNA pyrosequencing and iTRAQ-coupled LC-MS/MS.

After 454 pyrosequencing the sequences were clustered into 14,076 OTUs and 18 phyla, 28 classes, 48 orders, 78 families,135 genera, and 410 species were detected. These results exceeded the data of the previous HOMIM analysis of our group [30, 31]. The results of different sequencing technology methods could differ. To our knowledge, the HOMIM analysis has an emphasis on the predominant species of the bacterial community, while in the 16S rDNA pyrosequencing technology, the detection sensitivity of some species of bacteria is a little bit limited [17]. In the present study, 16S rDNA pyrosequencing could be more favorable to investigate a complete profile of the oral microbiome and discover some rare and non-cultivated bacteria that could be related to caries.

According to the results of alpha diversity indices, the richness and diversity of the bacterial communities in caries groups were similar to the caries-free group, as previously found in other studies [17, 19, 32]. However, Xiao et al., [18] demonstrated a higher bacterial diversity of healthy dental plaques compared to dental caries. These controversial results could be influenced by the difference between individuals, the selection process of subjects, sequencing technology methods, and other factors. Moreover, we found the six most abundant phyla including *Firmicutes, Bacteroidetes, Fusobacteria, Proteobacteria, Actinobacteria, and Candidate division TM7,* which were in agreement with the results of previous studies [13, 17, 18]. At the genus level, 135 genera were detected, including 13 prevalent genera, roughly similar to previous studies [32–34]. These dominant bacterial communities at the phylum and genus level were similar in the caries-free and caries-active sample, and merely the relative abundance was different. This indicates that the activity of specific microorganisms does not cause dental caries, some cariogenic bacteria are also part of the normal oral flora, and their presence is a constant variable [35]. *Dialister, Selenomonas, Actinomyces*, and *Mogibacterium* were identified at significantly higher levels in the caries-active sample using the LEfSe analysis, which could be recognized as a potential bacterial biomarker in dental caries. We speculated that changing some metabolic pathways and these bacteria’ biological characteristics are relate to caries in children .

At the beginning of caries, several microorganisms gather on the tooth surface in an ordered way, and then the oral ecosystem is broken when caries occur. Acidogenic and acid-tolerating species shift toward community dominance [36, 37]. In the current study, a higher abundances of Actinomyces were observed in the PH group compared to PN (*P* < 0.05), with the detection rates of Actinomyces odontolyticus being the highest. Actinomyces viscosus, an acid-producing bacterium associated with biofilm formation, was significantly higher in both SH and PH than in the caries-free group with low abundance. This indicated that some low abundances also could play an important role in in the oral microenvironment [25]. Actinomyces gerencseriae was higher in the caries-active group, which might be meaningful to investigate the correlation with caries in future studies. The Streptococcus genus had no significant difference between the caries-free and caries-active group. At the species level, Streptococcus sanguinis was higher in caries-free plaque than caries-active plaque. These outcomes could derive from different categories of severity. Streptococcus sanguinis settle on the tooth surface during early caries lesions and its population decrease with the development of caries [38]. The detection rates of Streptococcus mutans were lower than 0.2%, and it was significantly higher in the SH and PH group compared with the caries-free group. It is widely recognized that Streptococcus mutans is an acidogenic and aciduric bacterial species interrelated with caries. However, previous studies proved that caries occurred without the presence of Streptococcus mutans [8, 39]. The ecological plaque hypothesis emphasizes that the occurrence and development of dental caries result from an ecological imbalance between tooth mineral and microbial flora, and the upsurge in the acidogenic and aciduric component in the oral microenvironment would break the balance [9]. The current study demonstrated that the diversity of the microbial community has little effect on caries and some rarely detected bacteria but at higher levels in the caries-active sample would play a critical role in caries development, supporting the “ecological plaque hypothesis.”

Oral health and disease are correlated with the interplay inside the oral microbial community. Saliva, as the main microenvironment of oral bacteria, is considered a significant influence on the colonization of microorganisms [40]. The result of PCoA analysis revealed clear segregation between samples from dental plaque and saliva, meaning the distribution of microorganism structures in plaque were different from those of saliva. Ren et al., [13] suggested that dental plaque had significant phylogenetic differences compared with saliva and tongue coating. The reasons for this situation are probably related to the physicochemical features at different sites, such as pH, oxygen concentration, and bacterial adherence [41].

Human microbiological studies support the concept of a “core microbiome,” which is referred to the microbiome shared by most individuals in a specific environment of the body such as the skin, nasal cavity, intestinal tract, and oral cavity [42–45]. In our study, the Venn diagram shows that 52.6% of all the genera were shared and 18 predominant genera uniform was identified in saliva and plaque subjects, indicating the existence of “oral core microbiome,” as suggested by a previous study [18]. The core microbiome contributes to the functional stability and microecological balance of a healthy oral cavity.

For the result of salivary proteome analysis, we detected differentially expressed proteins and their functional classification between the SN and SH groups. Compared with the method of electrospray ionization ion-trap tandem mass spectrometry (ESI-MS/MS) used in our previous study, the number of proteins and peptides identified in our present study was higher [46]. Two hundred and fifty-eight proteins were found to be differentially expressed, which might play a part in the process of childhood dental caries. Some important proteins were included in differentially expressed proteins, such as *lactoferrin, matrix metalloproteinase-9, cystatin-B, mucin-7, protein S100-A9, proline-rich protein and so on*, which have demonstrated a potential relationship with caries in previous studies [47–49]. Lactoferrin is an antibacterial protein with the iron-chelating property directly binding to bacteria and agglutinate *S. mutans*. The combined bacteria are easy to be removed with the mechanical saliva action [50, 51]. Also, it was reported that there was a high correlation between matrix metalloproteinase-9 and caries lesion depth [49]. MMPs and cysteine cathepsins could affect the caries process in the early phases of demineralization [52]. The result of the GO analysis shows that differentially expressed proteins were associated with metabolic process and regulation of the biological process, mainly in the protein binding. As common salivary proteins, mucin-7 binding to proline-rich protein could be adsorbed onto the tooth surface to form a pellicle that regulates the bacteria adhesion and modulate the demineralization/remineralization process [53, 54]. Particularly, *azurocidin* was identified in the differentially expressed proteins, which has been found to be associated with gingivitis and early inflammatory periodontal destruction, and is a potential biomarker for periodontitis. But its possible anti-caries effect is worth further exploration. In addition, there were other proteins that were detected in differentially expressed in SH group or SN group, and their potential to cause or prevent caries needs to be further confirmed. The molecular sequencing techniques make precise identification of proteins. However, because of the complexity of saliva and immature technologies, proteinic information in our current research is not complete, and some low abundance proteins from the microorganism and its metabolite were not explored. The specific mechanism and more detailed information about the proteins in the saliva need to be further investigated. There is still a long way to devise strategies that modulate interactions of microbiota and salivary proteins for the treatment of oral diseases.

## Conclusion

In conclusion, alpha diversity analysis demonstrated that the richness and diversity of the bacterial communities were similar between caries and caries-free children. Then the PCoA analysis revealed segregation between the caries microbiota and saliva microbiota. Meanwhile, LEfSe analysis detected several bacteria at significantly higher levels in the caries-active sample, which could be recognized as a potential bacterial biomarker. A portion of the detected microorganisms was shared in all the samples, supporting the existence of an oral core microbiome. These bacteria play a critical role in keeping the balance of the oral microbial ecosystem. In salivary protein, we identified the differentially expressed proteins, as well as their functional classification. Using the iTRAQ technique, 258 proteins were found to be differentially expressed. These differentially expressed proteins could be associated with caries or health status, but more proteinic information should be further developed.

## Supplementary information

**Additional file 1: Table S1.** The SCX gradient information Showing SCX Gradient of fractionation.

**Additional file 2: Table S2.** The parameters used for the database search in Mascot search engine showing the parameters used for the database search.

**Additional file 3: Fig. S1**. Chao and Shannon curves of each group. **(a) (b)** represent the Chao curves of plaque and saliva group respectively; **(c) (d)** represent the Shannon curves of plaque and saliva group respectively.

**Additional file 4: Fig. S2.** Rarefaction curves of each group. **(a)** represent plaque group and **(b)** represent saliva group

**Additional file 5: Table S3.** The differential genera between SH and SN group. The black body represents the dominant bacteria (relative abundance > 1%).

**Additional file 6: Table S4.** The differential genera between PH and PN group. The black body represents the dominant bacteria (relative abundance > 1%).

**Additional file 7: Fig. S3** The difference of Actinomyces at species level. Each column one species of the Actinomyces.**(H1-H10)(N1-N10)(H11-H20)(N11-N20)** represent SH, SN,PH and PN, respectively.

**Additional file 8: Fig. S4.** The difference of Streptococcus at species level. Each column one species of the Streptococcus. **(H1-H10)(N1-N10)(H11-H20)(N11-N20)** represent SH, SN,PH and PN, respectively.

**Additional file 9: Fig. S5.** The nonmetric multidimensional scaling (NMDS) analysis. A dot represents each sample. **(H1-H10)(N1-N10)(H11-H20)(N11-N20)** represent SH, SN,PH and PN, respectively.

**Additional file 10: Fig. S6.** The SDS-PAGE electrophoresis of each group. Whole saliva from SN and SH were separated by SDS-PAGE.

**Additional file 11: Fig. S7.** Scatter plot of the Pearson Correlation. **(a)** represent SN group and **(b)** represent SH group.

**Additional file 12: Table S5.** Some differential protein between caries and healthy saliva.

**Additional file 13:.** The quantitative information of identified 1626 proteins.

## Data Availability

The datasets used during the current study are available from the corresponding author on reasonable request.

## Abbreviations

dmft: missing (due to caries), or filled tooth surfaces in primary teeth
iTRAQ: isobaric tags for relative and absolute quantitation
LC-MS/MS: Quantitative nano-flow liquid chromatography-tandem mass spectrometry
OUTs: Operational taxonomic units
PCoA: Principal coordinates analysis
FASP: Filter aided proteome preparation
GO: Gene ontology
LEfSe: Linear discriminant analysis of effect size
SN: Caries-free saliva group
PN: Caries-free plaque group
SH: Caries-active saliva group
PH: Caries-active plaque group
