# Supplementary material for: The oral microbiome and salivary proteins influence caries in children aged 6 to 8 years
Source: BMC Oral Health. 2020 Oct 28;20:295. doi: 10.1186/s12903-020-01262-9 (PMC7592381; doi:10.1186/s12903-020-01262-9)
Supplement: Supplementary file 1 — Additional file 1: Table S1. The SCX gradient information Showing SCX Gradient of fractionation. [file 12903_2020_1262_MOESM1_ESM.docx]

TableS1 SCX Gradient:

| Time (min) | %A | %B | Flowrate (ul/min) |
| --- | --- | --- | --- |
| 0.00 | 100 | 0 | 1000 |
| 25.00 | 100 | 0 | 1000 |
| 25.01 | 100 | 0 | 1000 |
| 32.00 | 90 | 10 | 1000 |
| 32.01 | 90 | 10 | 1000 |
| 42.00 | 80 | 20 | 1000 |
| 42.01 | 80 | 20 | 1000 |
| 47.00 | 55 | 45 | 1000 |
| 47.01 | 55 | 45 | 1000 |
| 52.00 | 0 | 100 | 1000 |
| 60.00 | 0 | 100 | 1000 |
| 60.01 | 100 | 0 | 1000 |
| 75.00 | 100 | 0 | 1000 |
