# Supplementary material for: The oral microbiome and salivary proteins influence caries in children aged 6 to 8 years
Source: BMC Oral Health. 2020 Oct 28;20:295. doi: 10.1186/s12903-020-01262-9 (PMC7592381; doi:10.1186/s12903-020-01262-9)
Supplement: Supplementary file 2 — Additional file 2: Table S2. The parameters used for the database search in Mascot search engine showing the parameters used for the database search. [file 12903_2020_1262_MOESM2_ESM.docx]

Table S2 parameters used for the database search in Mascot search engine

| Item | Value |
| --- | --- |
| Type of search | MS/MS Ion search |
| Enzyme | Trypsin |
| Mass Values | Monoisotopic |
| Max Missed Cleavages | 2 |
| Fixed modifications | Carbamidomethyl (C)，  Itraq4plex (N-term)，iTRAQ4plex (K) |
| Variable modifications | Oxidation (M)，iTRAQ4plex (Y) |
| Peptide Mass Tolerance | ± 20 ppm |
| Fragment Mass Tolerance | 0.1Da |
| Protein Mass | Unrestricted |
| Database | ipi.human.v3.87.fasta |
| Database pattern | decoy |
