# Supplementary figures and images for: The oral microbiome and salivary proteins influence caries in children aged 6 to 8 years
Source: BMC Oral Health. 2020 Oct 28;20:295. doi: 10.1186/s12903-020-01262-9 (PMC7592381; doi:10.1186/s12903-020-01262-9)

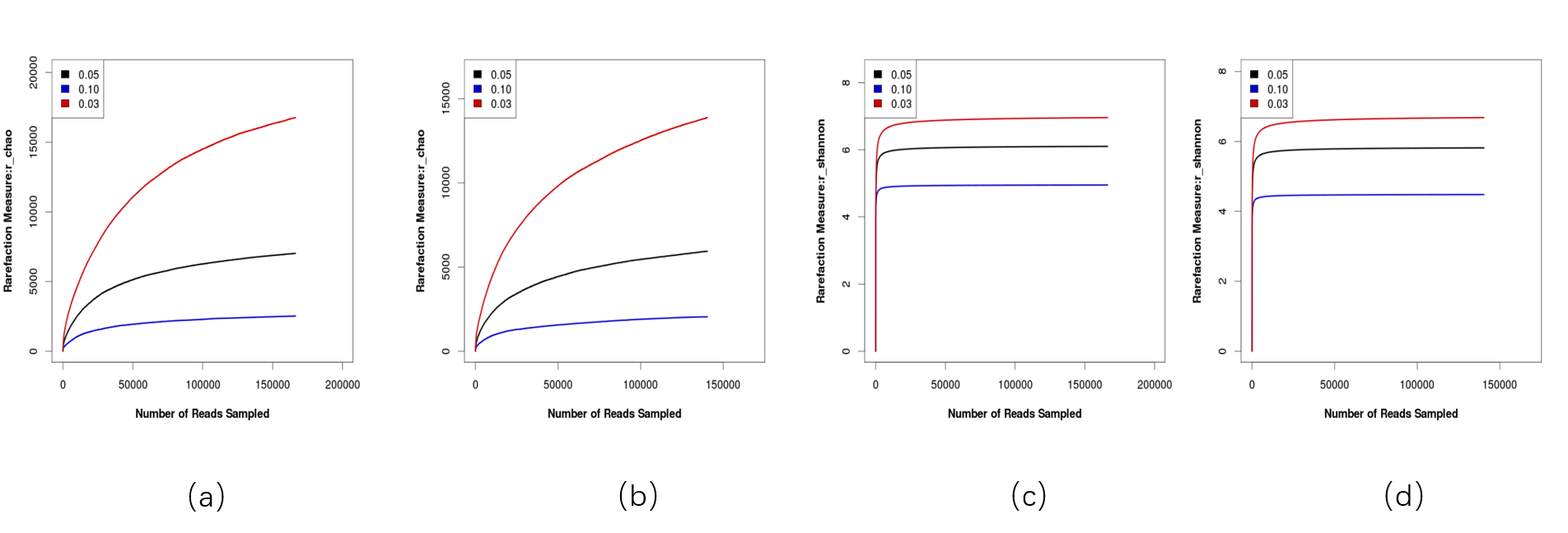

Supplement: Supplementary file 3 — Additional file 3: Fig. S1. Chao and Shannon curves of each group. (a) (b) represent the Chao curves of plaque and saliva group respectively; (c) (d) represent the Shannon curves of plaque and saliva group respectively. [file 12903_2020_1262_MOESM3_ESM.png]

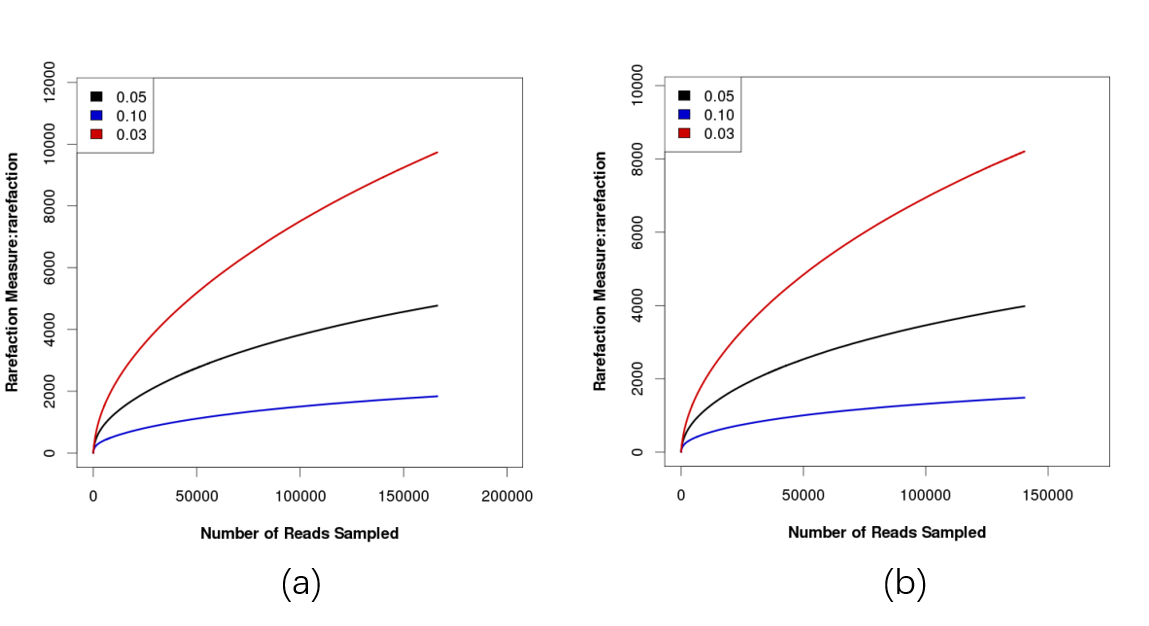

Supplement: Supplementary file 4 — Additional file 4: Fig. S2. Rarefaction curves of each group. (a) represent plaque group and (b) represent saliva group [file 12903_2020_1262_MOESM4_ESM.png]

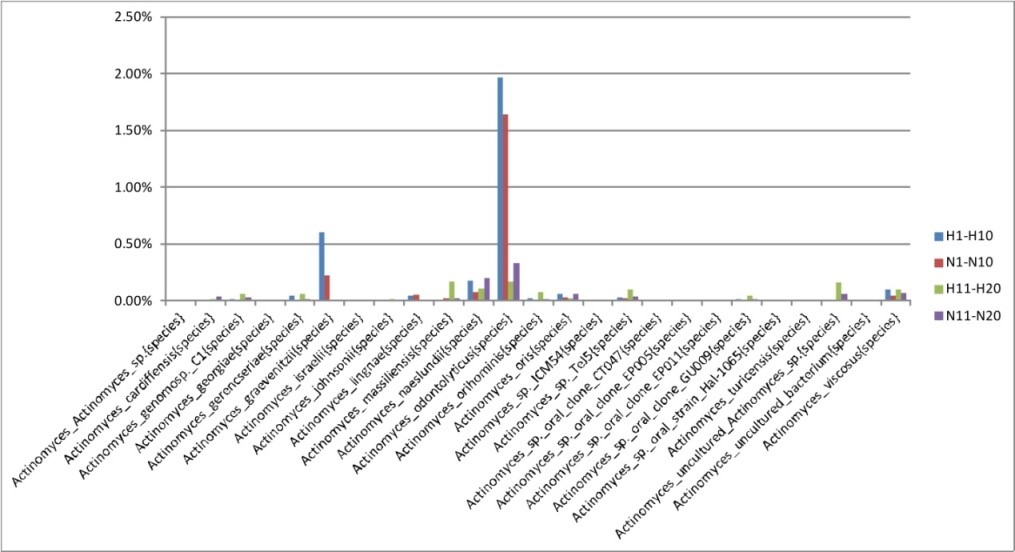

Supplement: Supplementary file 7 — Additional file 7: Fig. S3 The difference of Actinomyces at species level. Each column one species of the Actinomyces.(H1-H10)(N1-N10)(H11-H20)(N11-N20) represent SH, SN,PH and PN, respectively. [file 12903_2020_1262_MOESM7_ESM.jpg]

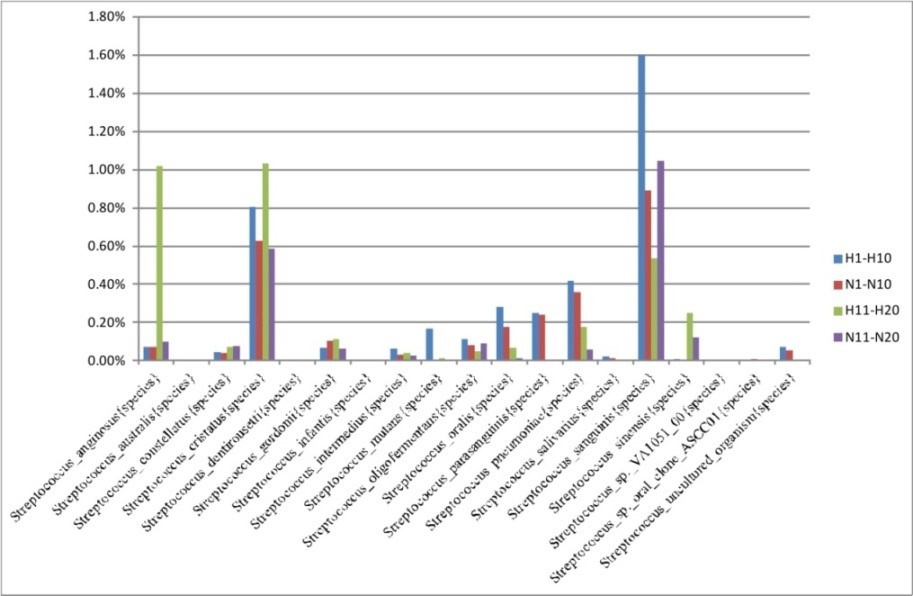

Supplement: Supplementary file 8 — Additional file 8: Fig. S4. The difference of Streptococcus at species level. Each column one species of the Streptococcus. (H1-H10)(N1-N10)(H11-H20)(N11-N20) represent SH, SN,PH and PN, respectively. [file 12903_2020_1262_MOESM8_ESM.jpg]

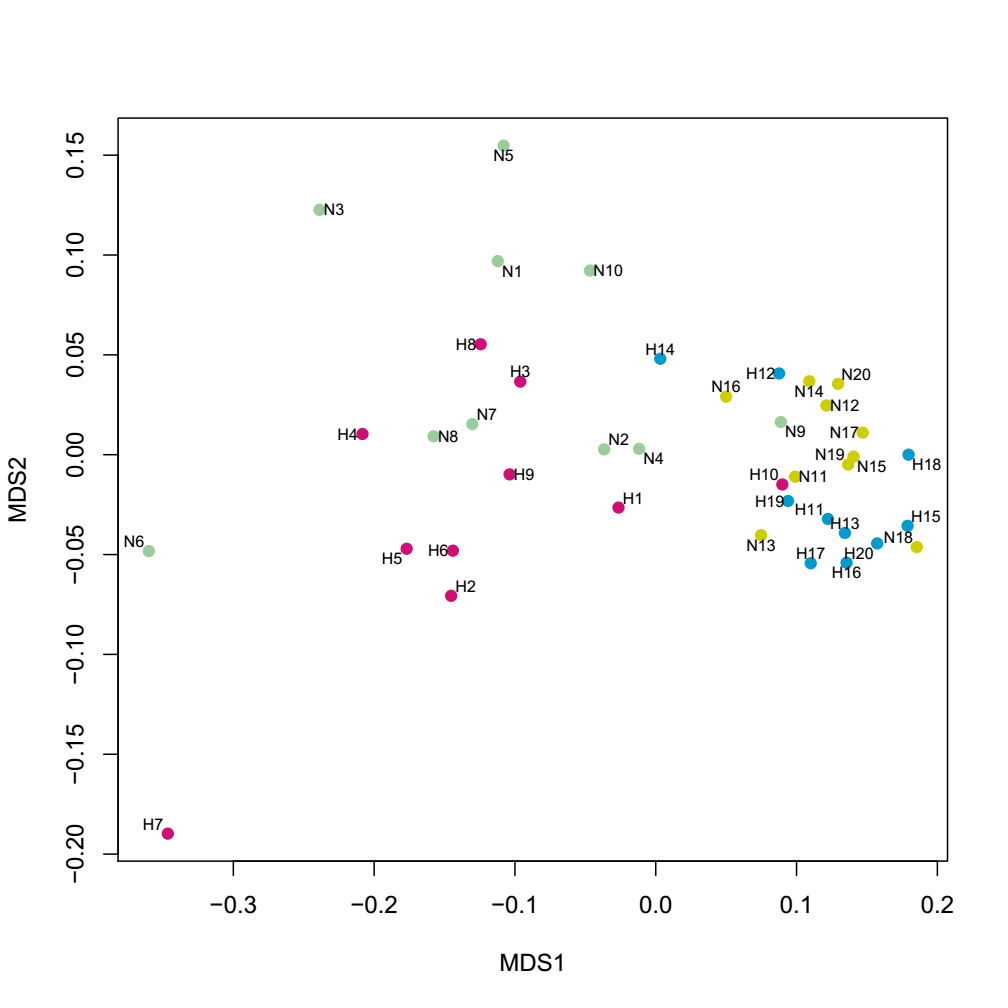

Supplement: Supplementary file 9 — Additional file 9: Fig. S5. The nonmetric multidimensional scaling (NMDS) analysis. A dot represents each sample. (H1-H10)(N1-N10)(H11-H20)(N11-N20) represent SH, SN,PH and PN, respectively. [file 12903_2020_1262_MOESM9_ESM.png]

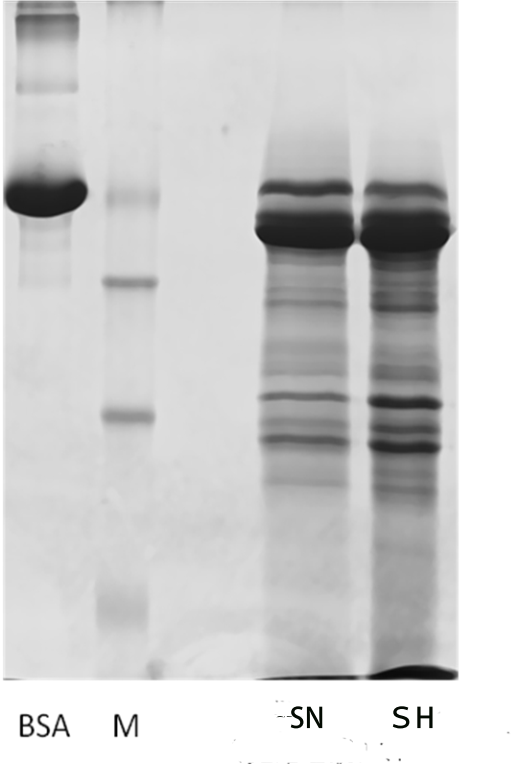

Supplement: Supplementary file 10 — Additional file 10: Fig. S6. The SDS-PAGE electrophoresis of each group. Whole saliva from SN and SH were separated by SDS-PAGE. [file 12903_2020_1262_MOESM10_ESM.png]

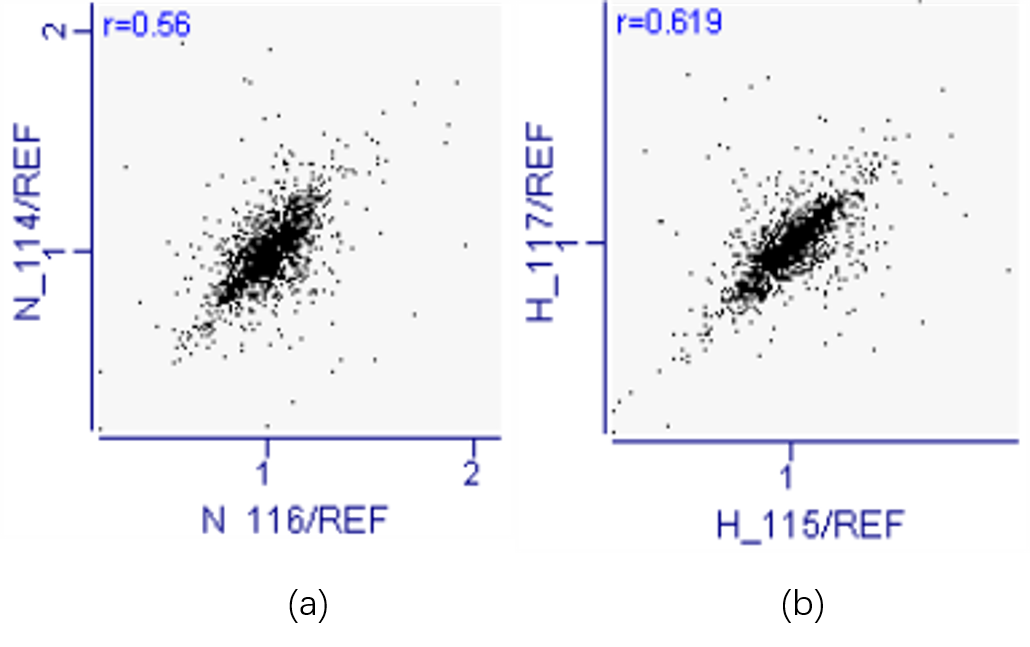

Supplement: Supplementary file 11 — Additional file 11: Fig. S7. Scatter plot of the Pearson Correlation. (a) represent SN group and (b) represent SH group. [file 12903_2020_1262_MOESM11_ESM.png]
