# Supplementary material for: The oral microbiome and salivary proteins influence caries in children aged 6 to 8 years
Source: BMC Oral Health. 2020 Oct 28;20:295. doi: 10.1186/s12903-020-01262-9 (PMC7592381; doi:10.1186/s12903-020-01262-9)
Supplement: Supplementary file 5 — Additional file 5: Table S3. The differential genera between SH and SN group. The black body represents the dominant bacteria (relative abundance > 1%). [file 12903_2020_1262_MOESM5_ESM.docx]

**TableS3 The differential genera between SH and SN group**

|  | SH | | SN | |  |
| --- | --- | --- | --- | --- | --- |
| genera | Mean | SD | Mean | SD | P value |
| Acholeplasma | 0 | 0 | 0.000104 | 0.000075 | 0.001594 |
| Acinetobacter | 0 | 0 | 0.000058 | 0.000031 | 0.027916 |
| Actinobaculum | 0.005386 | 0..002078 | 0.000872 | 0.00036 | 0.020979 |
| Alysiella | 0.000032 | 0.000021 | 0.000106 | 0.000072 | 0.040129 |
| Butyrivibrio | 0.000611 | 0.000234 | 0.000195 | 0.000073 | 0.046893 |
| Delftia | 0.000078 | 0.000031 | 0.000410 | 0.000144 | 0.044955 |
| **Derxia** | **0.016199** | **0.004441** | **0.007877** | **0.00192** | **0.044925** |
| Dialister | 0.000873 | 0.000350 | 0.000209 | 0.000101 | 0.040939 |
| Haemophilus | 0.002973 | 0.000794 | 0.005014 | 0.000933 | 0.043876 |
| Lactobacillus | 0.000298 | 0.000216 | 0 | 0 | 0.008991 |
| **Porphyromonas** | **0.048816** | **0.006439** | **0.087886** | **0.01963** | **0.043956** |
| Propionivibrio | 0.000156 | 0.000085 | 0.000025 | 0.000017 | 0.039127 |
| Pseudoramibacter | 0.000139 | 0.000084 | 0 | 0 | 0.001996 |
| Streptobacillus | 0.001783 | 0.000552 | 0.000727 | 0.000240 | 0.043896 |
| Coriobacteriaceae_uncultured | 0.000162 | 0.000086 | 0 | 0 | 0.003975 |
| Olsenella_sp._oral_taxon_809_str._F0356 | 0.000059 | 0.000059 | 0 | 0 | 0.062806 |
| Olsenella | 0.000081 | 0.000071 | 0 | 0 | 0.015785 |

**The black body represents the dominant bacteria(relative abundance >1%).**
