# Supplementary material for: The oral microbiome and salivary proteins influence caries in children aged 6 to 8 years
Source: BMC Oral Health. 2020 Oct 28;20:295. doi: 10.1186/s12903-020-01262-9 (PMC7592381; doi:10.1186/s12903-020-01262-9)
Supplement: Supplementary file 6 — Additional file 6: Table S4. The differential genera between PH and PN group. The black body represents the dominant bacteria (relative abundance > 1%). [file 12903_2020_1262_MOESM6_ESM.docx]

**Table****S4 The differential genera between PH and** **PN group**

|  | PH | | PN | |  |
| --- | --- | --- | --- | --- | --- |
| genera | Mean | SD | Mean | SD | P value |
| Actinomyces | 0.020176 | 0.002449 | 0.013913 | 0.001527 | 0.037962 |
| Bergeyella | 0.002163 | 0.000395 | 0.004348 | 0.001126 | 0.041938 |
| **Capnocytophaga** | **0.048747** | **0.005111** | **0.077051** | **0.00798** | **0.018981** |
| Cardiobacterium | 0.001046 | 0.000259 | 0.002274 | 0.000617 | 0.047922 |
| Derxia | 0.012829 | 0.002879 | 0.027195 | 0.006359 | 0.063936 |
| Granulicatella | 0.004289 | 0.000779 | 0.006984 | 0.001479 | 0.041878 |
| Haemophilus | 0.000196 | 0.000093 | 0.002497 | 0.001469 | 0.020979 |
| Kingella | 0.001997 | 0.000472 | 0.004869 | 0.001411 | 0.046933 |
| Megasphaera | 0.000490 | 0.000230 | 0.000077 | 0.000051 | 0.043896 |
| Mogibacterium | 0.000715 | 0.000242 | 0.000167 | 0.000070 | 0.028971 |
| Moraxella | 0.000036 | 0.000025 | 0.000415 | 0.000179 | 0.031968 |
| Neisseria | 0.036139 | 0.008708 | 0.069202 | 0.017078 | 0.041898 |
| Slackia | 0.000119 | 0.000088 | 2.11E-06 | 2.11E-06 | 0.042927 |
| Coriobacteriaceae_uncultured | 0.000267 | 0.000125 | 0.000025 | 0.000015 | 0.040909 |
| Veillonellaceae_uncultured | 0.000487 | 0.000302 | 0.000052 | 0.000040 | 0.048921 |

**The black body represents the dominant bacteria(relative abundance >1%).**
