# Supplementary material for: The oral microbiome and salivary proteins influence caries in children aged 6 to 8 years
Source: BMC Oral Health. 2020 Oct 28;20:295. doi: 10.1186/s12903-020-01262-9 (PMC7592381; doi:10.1186/s12903-020-01262-9)
Supplement: Supplementary file 12 — Additional file 12: Table S5. Some differential protein between caries and healthy saliva. [file 12903_2020_1262_MOESM12_ESM.docx]

**Table** **S5 Some differential protein between caries and healthy saliva**

| Actin | Cystatin-B | Lymphocyte antigen 6D |
| --- | --- | --- |
| adenosine kinase | Cystatin-D | Lysozyme C |
| adenosylhomocysteinase | Cystatin-S | Mammaglobin-B |
| Adenylyl cyclase-associated protein | Cystatin-SN | Matrix metalloproteinase-9,MMP9 |
| Alanyl-tRNA synthetase | Desmoglein-1 | Metalloproteinase inhibitor 2 |
| Alcohol dehydrogenase [NADP+] | Desmoglein-3 | Moesin |
| Aldehyde oxidase | Dipeptidyl peptidase 4 | MUC1 |
| Alpha-2-macroglobulin | Elafin | Mucin-16 |
| Angiotensinogen | Envoplakin | Mucin-5AC |
| Annexin A1 | Eosinophil cationic protein | Mucin-7 |
| Annexin A5 | Flavin reductase | Neutrophil elastase |
| Anti-folate binding protein | Furin | Nucleobindin-1 |
| Anti-mucin1 | Galectin-3 | Pentraxin-related protein PTX3 |
| Antithrombin-III | Galectin-3-binding protein | Peroxiredoxin-2 |
| Apolipoprotein A-IV | Galectin-7 | Phosphoglycerate kinase 1 |
| Apolipoprotein C | Glucose-6-phosphate isomerase | Phospholipase |
| Apolipoprotein D | Glutathione S-transferase P | Plasminogen |
| Apolipoprotein M | Glutathione synthetase | Plasminogen activator inhibitor 2 |
| Azurocidin | Haptoglobin | Plastin-2 |
| Beta-hexosaminidase | Heat shock factor-binding protein 1 | Plexin-B2 |
| Beta-mannosidase | Heat shock protein beta-1 | Prolactin-inducible protein |
| Calicin | Heat shock protein HSP 90-beta | Proline-rich protein 1 |
| Calmodulin | Hemoglobin | Prostaglandin-H2 D-isomerase |
| Calmodulin | Hemopexin | Proteasome |
| Calnexin | Heparin cofactor 2 | Protein S100-A11 |
| Calpain-1 | hepatoma-derived growth factor | Protein S100-A6 |
| calponin-2 | Hexokinase-3 | Protein S100-A7A |
| Calreticulin | Histatin-1 | Protein S100-A8 |
| Carbonic anhydrase 1 | Histidine-rich glycoprotein | Protein S100-A9 |
| Carbonic anhydrase 2 | Histone H2A | Resistin |
| Carboxypeptidase | Histone H2B | Rheumatoid factor D5 heavy chain |
| Caspase-14 | Ig heavy chain | Rho GDP-dissociation inhibitor 2 |
| Catalase | Ig kappa chain | Rho GTPase-activating protein 1 |
| Cathepsin B | Ig lambda chain | Secretoglobin family 3A member 2 |
| Cathepsin D | IGL@ protein | Serine/threonine-protein kinase OSR1 |
| Cathepsin G | Intelectin-1 | Serotransferrin |
| Cathepsin L1 | Involucrin | Sialic acid-binding Ig-like lectin 9 |
| Lactotransferrin | Complement factor H | Synaptotagmin-like protein 5 |
| Mucin-5B | Core histone macro-H2A | Thioredoxin |
| Ceruloplasmin | Myosin-9 | Thrombospondin-1 |
| Coactosin-like protein | Serpin B3 | Thymosin beta-4-like protein 3 |
| Collagen alpha-1(VI) chain | Enolase-phosphatase E1 | Transcobalamin-1 |
| Complement C1 | glycoprotein glucosyltransferase 1 | Transketolase |
| Complement C3 | Serine protease inhibitor Kazal | Trefoil factor 3 |
| complement C4 | Fibrinogen gamma chain | Trypsin-1 |
| Complement component C8 | Myeloperoxidase | Tubulin beta-2C chain |
| Complement component C9 | Tyrosine-protein kinase Lyn | Tyrosine-protein kinase CSK |
| Complement factor I | Kallikrein-14 | Vitronectin |
| Cornifin-A | Ladinin-1 | Zymogen granule protein 16 homolog B |
| Cornulin | Laminin |  |
| Cystatin-A | Lipocalin-1 |  |
